# The retina as a window into detecting subclinical cardiovascular disease in type 2 diabetes

**DOI:** 10.1038/s41598-025-13468-4

**Published:** 2025-07-31

**Authors:** Abbas S. Alatrany, Kishan Lakhani, Alice C. Cowley, Jian L. Yeo, Abhishek Dattani, Sarah L. Ayton, Aparna Deshpande, Matthew P.M. Graham-Brown, Melanie J. Davies, Kamlesh Khunti, Thomas Yates, Stephanie L. Sellers, Huiyu Zhou, Emer M. Brady, Jayanth R. Arnold, James Deane, Rebecca J. McLean, Frank A. Proudlock, Gerry P. McCann, Gaurav S. Gulsin

**Affiliations:** 1 Department of Cardiovascular Sciences, National Institute for Health Research Leicester Biomedical Research Centre and British Heart Foundation Centre of Research Excellence, University of Leicester, Leicester, UK; 2 Department of Imaging Services, University Hospitals of Leicester NHS Trust, Leicester, UK; 3 Department of Renal Medicine, University Hospitals of Leicester NHS Trust, Leicester, UK; 4 Leicester Diabetes Centre, University of Leicester and the NIHR Leicester Biomedical Research Centre, Leicester, UK; 5 Cardiovascular Translational Laboratory, St Paul’s Hospital, University of British Columbia Centre for Heart Lung Innovation, Vancouver, BC Canada; 6 School of Computing and Mathematical Sciences, University of Leicester, Leicester, UK; 7 The University of Leicester Ulverscroft Eye Unit, Leicester Royal Infirmary, Leicester, UK; 8 Department of Cardiovascular Sciences, NIHR Leicester Biomedical Research Centre, University of Leicester, Glenfield Hospital, Groby Road, Leicester, LE3 9QP UK

**Keywords:** Cardiovascular disease, Type 2 diabetes, Digital retinal photography, Diabetic cardiomyopathy, Atherosclerotic coronary artery disease, Heart failure., Biomarkers, Predictive markers, Cardiology, Diabetes

## Abstract

Individuals with Type 2 Diabetes (T2D) are at high risk of subclinical cardiovascular disease (CVD), potentially detectable through retinal alterations. In this single-centre, prospective cohort study, 255 asymptomatic adults with T2D and no prior history of CVD underwent echocardiography, non-contrast coronary computed tomography and cardiovascular magnetic resonance. Retinal photographs were evaluated for diabetic retinopathy grade and microvascular geometric characteristics using deep learning (DL) tools. Associations with cardiac imaging markers of subclinical CVD were explored. Of the participants (aged 64 ± 7 years, 62% males); 200 (78%) had no diabetic retinopathy and 55 (22%) had mild background retinopathy. Groups were well-matched for age, sex, ethnicity, CV risk factors, urine microalbuminuria, and serum natriuretic peptide and high-sensitivity troponin levels. Presence of retinopathy was associated with a greater burden of coronary atherosclerosis (coronary artery calcium score ≥ 100; OR 2.63; 95% CI 1.29–5.36; *P* = 0.008), more concentric left ventricular remodelling (OR 3.11; 95% CI 1.50–6.45; *P* = 0.002), and worse global longitudinal strain (OR 2.32; 95% CI 1.18–4.59; *P* = 0.015), independent of key co-variables. Early diabetic retinopathy is associated with a high burden of coronary atherosclerosis and markers of early heart failure. Routine diabetic eye screening may serve as an effective alternative to currently advocated screening tests for detecting subclinical CVD in T2D, presenting opportunities for earlier detection and intervention.

**Supplementary Information:**

The online version contains supplementary material available at 10.1038/s41598-025-13468-4.

## Introduction

Type 2 diabetes (T2D) is an established risk factor for cardiovascular disease (CVD)^1^. A high proportion of people with T2D have asymptomatic coronary artery disease or cardiac structural and/or functional alterations (termed *stage B heart failure*), which are markers of substantially increased downstream CV risk and mortality^1^. Screening for subclinical CVD with annual serum natriuretic peptide and high-sensitivity cardiac troponin is now advocated for early detection^2^. However, natriuretic peptide levels are influenced by factors common in T2D (e.g. obesity, increasing age, renal dysfunction, and use of renin angiotensin aldosterone inhibitors), compromising reliability^3^. Similarly, high-sensitivity cardiac troponins lack sufficient sensitivity and specificity to detect asymptomatic coronary disease or stage B heart failure^4^. A more robust tool to screen for subclinical CVD in T2D may permit earlier identification, risk stratification, and targeted management.

Diabetic retinopathy has consistently been associated with a higher risk of developing CVD^5^. However, few studies have comprehensively explored links between presence of retinopathy and underlying coronary disease and cardiac structural and functional alterations^6,7^. In the UK, diabetic retinopathy screening is routinely performed at T2D diagnosis and annually thereafter using retinal photography. If paired with advanced non-invasive cardiac imaging techniques for cardiac structure, function, and perfusion, this offers a unique opportunity to detect subclinical CVD through alterations in the retinal microcirculation^8^.

The aims of this study were: (1) to explore associations between presence of diabetic retinopathy and subclinical CVD, and (2) determine whether DL based interrogation of eye screening digital retinal photographs could enhance detection of subclinical cardiac structural/functional alterations or coronary atherosclerosis typical of diabetic heart disease.

## Methods

### Study population

This is a retinal sub-study of the single centre, prospective cohort study: Prevalence and Determinants of Subclinical Cardiovascular Dysfunction in Adults with Type 2 Diabetes (PREDICT) (NCT03132129). Adults with T2D were prospectively enrolled from primary and specialist care services in Leicestershire, UK, with support from the NIHR East Midlands Clinical Research Network. Inclusion criteria were age 18 to 75 years, with no prior history, clinical signs or symptoms of CVD. Exclusion criteria were type 1 diabetes, stage 4 or 5 chronic kidney disease (estimated glomerular filtration rate < 30mL/min/1.73m^2^), known macrovascular disease (including myocardial infarction, transient ischemic attack, stroke, peripheral artery disease), presence of arrhythmia, history of heart failure, moderate or severe valvular heart disease, and CV symptoms (such as angina or limiting dyspnea during normal physical activity). Included in the current sub-study analyses were those participants with paired digital retinal photographs, obtained from the NHS Leicestershire and Rutland Diabetic Eye Screening Programme, within one year of their PREDICT study visit (detailed below).Ethical approval was granted by the National Research Ethics Service (17/WM/0192), and the study adhered to the principles of the Declaration of Helsinki. Written informed consent was obtained from all participants.

### Assessments

CV study assessments were performed during a single study phenotyping visit. Digital retinal photographs were performed at a separate date, within one year of the CV study assessments. 

#### Bio-anthropometrics

Demographics, medical history, and anthropometric measurements, presence of hypertension and/or hypercholesterolemia were obtained from history and by prescribed medications. Smoking status was categorized as “never smoked”, “ex-smoker”, or “current smoker”. Resting blood pressure was measured in clinic as previously described^9^. A fasting blood sample was collected for biochemical profiling including full blood count, liver function, renal function, lipid profile, N-terminal pro brain natriuretic peptide (NTproBNP), high sensitivity troponin I, and glycosylated hemoglobin (HbA1c). A urine specimen was collected for measurement of urine albumin creatinine ratio. All samples were analyzed in an accredited NHS pathology lab at the University Hospitals of Leicester NHS Trust.

#### Transthoracic echocardiography

Resting transthoracic echocardiography was performed for assessment of diastolic function and reported by accredited operators using an iE33b system with X5-1 transducer (Phillips Medical Systems, Best, Netherlands) as per the American Society of Echocardiography guidelines^10^.

#### Cardiovascular magnetic resonance imaging

Cardiovascular magnetic resonance imaging (CMR) was performed using a standardised protocol on 3T Siemens scanners (Skrya or Vida, Erlangen, Germany) as previously described^11^. Myocardial blood flow image acquisition was performed using a dual-sequence spoiled gradient echo method with inline automated reconstruction and post-processing^12^. Perfusion images were acquired following vasodilator stress with adenosine (140–210 µg/kg/min) infusion for 3–5 min. At peak stress, a gadolinium-based contrast agent (Dotarem^®^, 0.075mmol/kg) was injected followed by a 20mL bolus of normal saline, at a rate of 5mL/s. Perfusion images were acquired at three short-axis left ventricle (LV) planes (basal, mid-ventricular, and apical). Rest imaging was conducted approximately 10 min after stress. Between stress and rest perfusion imaging, a stack of short-axis cine slices covering the entire LV was obtained. Late gadolinium enhancement images were acquired at least five minutes after rest perfusion to assess silent myocardial infarct and focal myocardial fibrosis. Late gadolinium enhancement images were acquired in the same positions as long- and short-axes cine images, using a phase-sensitive inversion recovery reconstruction sequence. Pre- and post-contrast T1 mapping was performed with a Modified Look-Locker inversion recovery sequence (Siemens MyoMaps, Erlangen, Germany). Images were analyzed using cvi42 (Version 5.10.1, Circle Cardiovascular Imaging, Calgary, Alberta, Canada) by one of two trained observers blinded to participant demographic and clinical details as previously described^12^. LV mass to end-diastolic volume ratio (LVM/V) was calculated as a marker of LV concentric remodeling. LV strain and strain rates derived from cine-based feature-tracking were presented as absolute values, where lower values indicate worse myocardial mechanics^13^. Late gadolinium enhancement images were assessed qualitatively for focal fibrosis and categorized as “infarct”, “non-infarct”, or “mixed” pattern, based on presence and distribution, by two observers (G.P.M. and A.C.). Perfusion images were firstly assessed qualitatively (G.P.M.) for regional perfusion defects indicative of ischemia due to epicardial coronary disease as per clinical standards^14^. Global stress and rest myocardial blood flow, and myocardial perfusion reserve (derived as the ratio of stress to rest blood flow across all myocardial segments), were calculated inline as detailed above^13^. Myocardial extracellular volume fraction, a surrogate marker of diffuse interstitial fibrosis, was calculated from pre- and post-contrast T1 maps^15^.

#### Non-contrast cardiac computed tomography

For measurement of coronary artery calcium and epicardial adipose tissue (EAT) volume, non-contrast electrocardiogram-gated cardiac computed tomography scans were obtained using a 128-detector computed tomography scanner (Siemens Somatom Flash, Erlangen, Germany). Scans used prospective electrocardiographic triggering set at 70% R-R interval and a tube voltage of 120kVp. Raw data underwent reconstruction with a slice thickness of 3.0 mm. Coronary artery calcium (CAC) scoring was performed using the Agatston method^16^. Total EAT volume was measured using a fully automated DL software (QFat, version 2.0; Cedars-Sinai Medical Center, Los Angeles, California) as previously described^17^.

#### Digital retinal photography images

Digital retinal photography images were obtained from the NHS Leicestershire and Rutland Diabetic Eye Screening Programme (DESP) using OptoMize software, capturing fovea and optic disc-centred images for each eye, totalling four images per participant. Retinopathy grading was performed by DESP according to the UK National Screening Committee’s standardised protocols and assurance procedures. Grading was conducted independently and blinded to cardiovascular phenotyping data. Disagreements were resolved through internal adjudication by senior graders or ophthalmologists in accordance with DESP protocol^18^. Retinopathy was classified using the RxMx grading system as either no diabetic retinopathy or background diabetic retinopathy, with the highest grade recorded per participant. Due to the low prevalence of moderate or severe retinopathy (*n* = 4), these cases were excluded to avoid statistical bias.

**Fig. 1:**
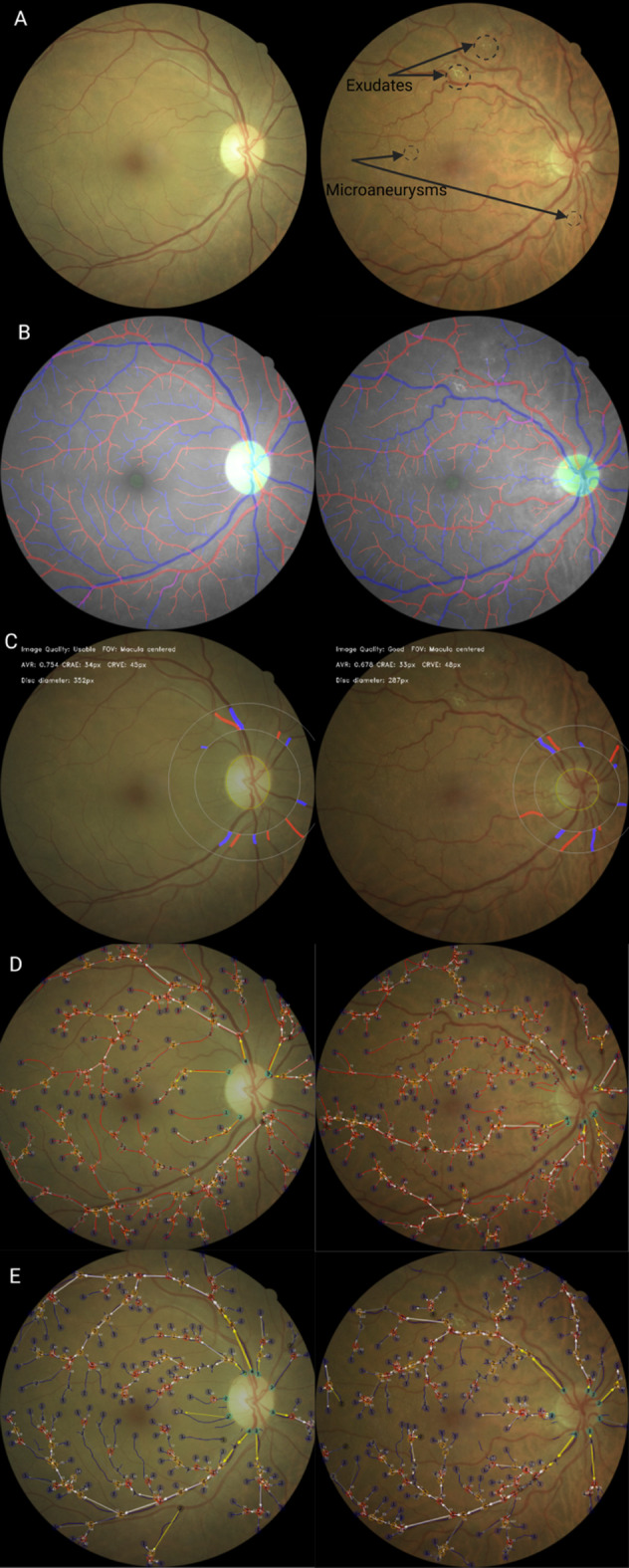
Retinal Microvascular Features. The left panel exhibits a retinal photograph with no diabetic retinopathy, the right panel exhibits a retinal photograph with background diabetic retinopathy. **A)** original retinal photograph; **B**) automatic segmentation; **C)** calculation of central retinal artery equivalent, central retinal venular equivalent, and arterio-venular ratio; **D**) automatic segmentation of arteries; **E)** automatic segmentation of veins.

Retinal microvascular geometry was quantified using an open-source, validated, fully automated vessel segmentation software (Retina-based Microvascular Health Assessment System)^19^. Using this, we derived: arterio-venular ratio (AVRE), central retinal arterial equivalent (CRAE), central retinal venular equivalent (CRVE), tortuosity, and fractal dimension. Figure 1 presents representative retinal images depicting diabetic retinopathy grades and microvascular quantification.

### Statistical analysis

Normality was assessed visually using histograms and Q-Q plots. Continuous data were expressed as mean (standard deviation) if normally distributed or median (25th – 75th percentile range) if not. Categorical variables were presented as count (%) and compared using Chi-squared test. Imaging parameters were compared between participants without and with background diabetic retinopathy using analysis of covariance adjusted for age, sex, ethnicity, body mass index, systolic blood pressure and HbA1c. The interval between digital retinal photography acquisition and CV assessment was recorded for all participants. A sensitivity analysis was conducted by stratifying the cohort into two groups: those with retinal imaging within 6 months of CV assessment and those with retinal imaging between 6–12 months from CV assessment. Group allocation accounted for both pre- and post-CV assessment dates. This analysis was conducted to determine the impact of temporal separation between retinal and cardiac imaging on the strength of observed associations.

Correlations between vascular geometric characteristics and cardiac imaging parameters were assessed using Pearson correlation coefficient in all subjects (with and without retinopathy). Multivariable associations between presence of retinopathy and imaging markers of subclinical CVD were explored using binomial logistic regression modeling. A base model was adjusted for age, sex, ethnicity, diabetes duration, BMI, systolic blood pressure, glycated hemoglobin and presence of retinopathy as covariables. Imaging markers of subclinical CVD (categorized as present or absent for each marker) were then individually added to this base model to determine independent associations with presence of retinopathy, presented as adjusted odds ratios and 95% confidence intervals. Cut-offs for defining subclinical CVD from cardiac imaging were defined a priori on the basis that they are either established markers of CAD^20^ or of stage B heart failure in T2D^21^ and included: total CAC ≥ 100 on non-contrast cardiac computed tomography, average E/e’ ≥15 (a non-invasive marker of LV filling pressure) on echocardiography^2^, and concentric LV remodelling (LVM/V ≥ 1.0)^22^ LV global longitudinal strain (GLS) < 16%^23^, coronary microvascular dysfunction (defined as myocardial perfusion reserve < 2.5)^24^, presence of regional ischemia and presence of LGE on CMR.

Standard statistical analyses were performed using Statistical Package for Social Services version 28.0 (SPSS Inc. Chicago, Illinois, USA). A p-value < 0.05 was considered statistically significant.

#### Deep learning-based prediction modelling

**Fig. 2:**
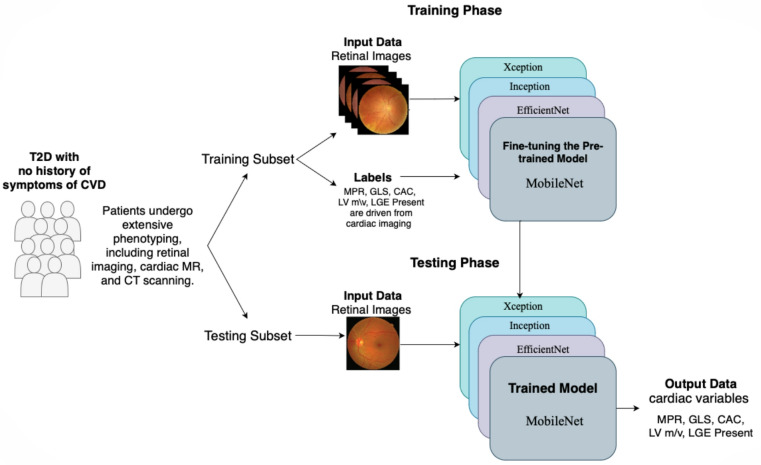
Overview of the deep learning workflow utilising transfer learning for detecting cardiovascular dysfunction from retinal photography. *Abbreviations*: CAC = coronary artery calcium score; GLS = global longitudinal strain; MPR = myocardial perfusion reserve; LGE = late gadolinium enhancement; LV M/V = left ventricular mass-to-volume ratio.

An overview of the DL workflow utilising transfer learning for detecting CV dysfunction from retinal photography is provided in Fig. 2. Four transfer learning models—Xception^25^, Inception^26^, EfficientNet^27^, and MobileNet^28^ - were selected to investigate the performance of their various architectures, with increasing complexity, in predicting imaging markers of subclinical CVD from paired retinal images in our dataset.

Transfer learning was applied to our dataset, with models fine-tuned during training to adapt to the task at hand. The dataset with split, with 80% allocation for training and 20% reserved for validation and testing.

To investigate the potential enhancement of model performance through the integration of clinical data to retinal images, we also employed a data fusion approach. Features from retinal images were extracted using a pretrained MobileNet model^28^, while clinical data were processed using an XGBoost model^29^. Late fusion was then applied to amalgamate both data types, facilitating comprehensive feature representation. For pre-processing clinical data, continuous variables were normalized using min-max normalization and categorical variables were encoding as either or one-hot based on data type. Missing values were addressed by either imputing the mode of the variable for categorical variables or the mean of the variable for continuous variables. Model performance was evaluated through accuracy, sensitivity, specificity, and area under the curve (AUC), with traditional machine learning models implemented in scikit-learn^30^, and transfer learning models developed in Keras with TensorFlow^31^.

## Results

### Baseline characteristics

An initial 259 subjects with complete CV phenotyping assessments and paired digital retinal photographs acquired within one year were enrolled. Four participants were excluded due to the presence of moderate or severe diabetic retinopathy, leaving a final sample of 255 subjects (1,020 retinal images) included in these analyses. All images met quality standards for diabetic retinopathy grading per national protocols. Among these, 200 (78%) had no diabetic retinopathy and 55 (22%) had background diabetic retinopathy.

Demographic, anthropometric, clinical and biochemical characteristics of study subjects stratified by diabetic retinopathy grade are shown in Table 1. Overall, mean age was 64 ± 7 years, 157 (62%) were males, 61 (24%) were of south Asian, Black, or other minority ethnic group, and mean body mass index 30.1 ± 5.1 kg/m^2^. Groups with and without diabetic retinopathy were of similar age, sex and ethnic distribution, had similar body mass index, blood pressure, and prevalence of medical comorbidities. Median diabetes duration was longer in those with versus without diabetic retinopathy, and a higher proportion of those with versus without diabetic retinopathy were on treatment with metformin and/or insulin. There were no differences in renal function, presence of micro- or macro-albuminuria, lipid profile, serum NTproBNP or high-sensitivity troponin levels, between groups with and without diabetic retinopathy.

**Table 1:** Baseline characteristics.

	All (*N* = 255)	No retinopathy (*N* = 200)	Mild background retinopathy (*N* = 55)
Age, years	64 ± 7	64 ± 7	62 ± 6
Male sex, n (%)	157 (62)	123 (62)	34 (62)
Ethnicity			
White European, n (%)	194 (76)	150 (75)	44 (80)
South Asian, n (%)	53 (21)	44 (22)	11 (20)
Black or other minority ethnic group, n (%)	8 (3)	8 (4)	0 (0)
Diabetes duration, years	9 (5–14)	8 (5–12)	13 (6–18)
Smoking status			
Never smoked, n (%)	142 (56)	114 (57)	28 (51)
Ex smoker, n (%)	93 (36)	69 (35)	24 (44)
Current smoker, n (%)	20 (8)	17 (8)	3 (5)
Hypertension, n (%)	149 (58)	114 (57)	35 (64)
Dyslipidaemia, n (%)	181 (71)	143 (56)	38 (69)
BMI, kg/m2	30.1 ± 5.1	30.2 ± 5.2	29.4 ± 4.6
Systolic BP, mmHg	135 ± 17	135 ± 17	133 ± 18
Diastolic BP, mmHg	81 ± 9	81 ± 9	80 ± 10
Heart rate, bpm			
ACE inhibitor, n (%)	98 (38)	74 (37)	24 (44)
ARB, n (%)	32 (13)	26 (13)	6 (11)
Beta blocker, n (%)	17 (7)	13 (7)	4 (7)
CCB, n (%)	64 (25)	52 (26)	12 (22)
Statin, n (%)	185 (73)	147 (74)	38 (69)
Insulin, n (%)	34 (13)	20 (10)	14 (25)
Metformin, n (%)	185 (73)	138 (69)	47 (85)
Sulphonylurea, n (%)	38 (15)	26 (13)	12 (22)
GLP-1RA, n (%)	24 (9)	17 (9)	7 (13)
SGLT2i, n (%)	57 (22)	42 (21)	15 (27)
Creatinine, umol/L	78 ± 18	78 ± 17	77 ± 17
eGFR, ml/min	84 (76–90)	84 (75–90)	85 (77–90)
Urine ACr mg/mmol	1.4 (0.6–2.8)	1.4 (0.6–2.9)	1.4 (0.6–2.6)
ACr category, n (%)			
No albuminuria (< 2.5)	147 (71)	112 (70)	35 (74)
A1 (2.5–3)	11 (5)	8 (5)	3 (6)
A2 (3–30)	42 (20)	34 (21)	8 (17)
A3 (> 30)	6 (3)	5 (3)	1 (2)
Total cholesterol, mmol/L	4.27 ± 0.96	4.31 ± 0.94	4.14 ± 1.04
LDL cholesterol, mmol/L	2.18 ± 0.76	2.22 ± 0.75	2.05 ± 0.77
HbA1c, %	7.3 ± 1.1	7.2 ± 1.1	7.5 ± 1.0
HbA1c, mmol/mol	56 ± 12	55 ± 12	58 ± 11
NTproBNP, pg/ml	44 (35–72)	45 (35–75)	39 (35–61)
NTproBNP ≥ 125 pg/ml, n (%)	22 (9)	19 (9)	3 (5)
hsTroponinI, ng/l	2.6 (2.5–4.2)	2.6 (2.5–4.2)	2.5 (2.5–4.5)

**Abbreviations**: ACE = angiotensin converting enyme; ACr = albumin-to-creatinine ratio; ARB = angiotensin receptor blocker; BMI = body mass index; BP = blood pressure; CCB = calcium channel blocker; GLP1-RA = glucagon-like peptide 1 receptor agonist; LDL = low density lipoprotein; SGLT2i = sodium glucose co-transporter 2 inhibitor.

### Cardiac imaging

Transthoracic echocardiography, non-contrast cardiac computed tomography and CMR imaging variables comparing those with and without diabetic retinopathy are presented in Table 2. Overall, those with background diabetic retinopathy had higher non-invasive LV filling pressures (average E/e’ 9.6 ± 2.5 vs. 9.0 ± 1.0; *P =* 0.012) and lower diastolic relaxation rates (LV circumferential peak early diastolic strain rate 0.81 ± 0.25 vs. 0.88 ± 0.24 s^− 1^; *P* = 0.02), higher burden of coronary atherosclerosis (total CAC 166 [21–510] vs. 21 [0–239] Agatston units; *P* < 0.001), more concentric LV remodelling (LVM/V 0.94 ± 0.15 vs. 0.90 ± 0.14 g/mL; *P* = 0.015), and poorer LV longitudinal shortening (GLS 15.2 ± 2.3 vs. 16.5 ± 2.3%; *P* < 0.001) despite similar LV ejection fraction, than those with no diabetic retinopathy. There were no differences in LV volumes, focal or diffuse fibrosis (late gadolinium enhancement, native T1 or extracellular volume fraction), or myocardial blood flow measures between groups.

**Table 2:** Cardiac imaging variables. Between group comparisons are adjusted for age, sex, ethnicity, body mass index, systolic blood pressure and HbA1c.

	All (*N* = 255)	No retinopathy (*N* = 200)	Mild background retinopathy (*N* = 55)	*Adjusted *P* value
**Echocardiography**				
E/A ratio	0.87 ± 0.19	0.87 ± 0.19	0.88 ± 0.19	0.968
Average E/e’	9.2 ± 2.1	9.0 ± 1.0	9.6 ± 2.5	**0.012**
**Non-contrast cardiac CT**				
Total calcium score	45 (0–279)	21 (0–239)	166 (21–510)	**< 0.001**
CAC = 0, n (%)	62 (26)	57 (30)	5 (9)	**0.013**
CAC 1-100, n (%)	81 (33)	63 (33)	18 (34)	
CAC 101–400, n (%)	53 (22)	37 (19)	16 (30)	
CAC > 400, n (%)	47 (19)	33 (17)	14 (26)	
Total EAT volume, cm3	132 ± 57	132 ± 59	129 ± 49	0.999
**Cardiac MRI**				
LV EDVi, ml/m2	63 ± 13	64 ± 13	62 ± 12	0.316
LV ESVi, ml/m2	22 ± 8	22 ± 8	22 ± 6	0.981
LV EF, %	67 ± 7	67 ± 7	65 ± 7	0.165
LVMi, g/m2	57 ± 10	56 ± 10	58 ± 9	0.232
LV M/V, g/ml	0.91 ± 0.14	0.90 ± 0.14	0.94 ± 0.15	**0.015**
Strain/strain rates				
LV GCS, %	19.4 ± 2.5	19.5 ± 2.6	19.1 ± 2.2	0.409
LV GLS, %	16.2 ± 2.4	16.5 ± 2.3	15.2 ± 2.3	**< 0.001**
LV circPEDSR, 1/s	0.86 ± 0.24	0.88 ± 0.24	0.81 ± 0.25	**0.02**
LV longPEDSR, 1/s	0.65 ± 0.19	0.66 ± 0.19	0.60 ± 0.19	**0.02**
Tissue characterisation				
Native T1, ms	1224 ± 37	1224 ± 36	1226 ± 40	0.793
ECV, % (available in 176)	27 ± 3	27 ± 3	27 ± 3	0.668
LGE present, n (%)	56 (22)	43 (22)	23 (24)	0.712
LGE infarct pattern, n (%)	10 (4)	6 (3)	4 (7)	0.342
LGE non-infarct, n (%)	45 (18)	36 (18)	9 (16)	
LGE mixed, n (%)	1 (0)	1 (1)	0 (0)	
Perfusion				
Perfusion defect, n (%)	21 (9)	14 (8)	7 (13)	0.206
Stress MBF, mL/min/g	1.82 ± 0.58	1.80 ± 0.58	1.87 ± 0.58	0.387
Rest MBF, mL/min/g	0.65 ± 0.18	0.66 ± 0.18	0.63 ± 0.18	0.245
Myocardial perfusion reserve	2.86 ± 0.88	2.81 ± 0.92	3.02 ± 0.76	0.127

*Abbreviations*: CAC = coronary artery calcium; EAT = epicardial adipose tissue; ECV = extracellular volume fraction; EDVi = end-diastolic volume indexed to body surface area; EF = ejection fraction; ESVi = end-systolic volume indexed to body surface area; GCS = global circumferential strain; GLS = global longitudinal strain; LGE = late gadolinium enhancement; LV = left ventricle; MBF = myocardial blood flow; Mi = mass indexed to body surface area; M/V = end-diastolic mass/volume; circPEDSR = circumferential peak early diastolic strain rate; longPEDSR = longitudinal peak early diastolic strain rate.

The median interval between retinal and CV imaging was 96 days (IQR: **42–174 days**; **Supplementary Table 2**). A sensitivity analysis stratifying participants by retinal imaging interval (≤ 6 months vs. 6–12 months; **Supplementary Table 3**) demonstrated that associations between background diabetic retinopathy and key markers of subclinical cardiovascular disease—including coronary artery calcium ≥ 100, impaired global longitudinal strain, and concentric LV remodelling—remained consistent across both groups.

### Geometric retinal analysis

Retinal microvascular geometric variables comparing subjects with and without diabetic retinopathy are displayed in Table 3. Those with background diabetic retinopathy had a wider CRVE compared to those without diabetic retinopathy. There were no significant differences between AVRE, CRAE, CRVE, fractal dimension or tortuosity between groups with and without diabetic retinopathy. A weak, but statistically significant association was observed between CRVE and LV M/V. No other significant correlations were observed between retinal microvascular geometric measures and pre-specified markers of subclinical CVD (Table 4).

**Table 3:** Comparison of retinal microvascular geometric variables between subjects with and without diabetic retinopathy.

Variable	No retinopathy (*N* = 200)	Mild background retinopathy (*N* = 55)	*P* value
AVRE	0.66 ± 0.05	0.64 ± 0.06	0.162
CRAE (µm)	166.63 ± 19.46	161.58 ± 20.77	0.094
CRVE (µm)	255.41 ± 32.60	251.86 ± 28.74	0.464
Fractal dimension	1.69 ± 0.03	1.69 ± 0.03	0.955
Tortuosity	1.09 ± 0.01	1.09 ± 0.01	0.826

*Abbreviations*: AVRE = arterio-venular ratio; CRAE = central retinal artery equivalent diameter; CRVE = central retinal venular equivalent diameter.

**Table 4:** Corelations between retinal microvasculature geometry and key markers of subclinical cardiovascular disease.

	AVRE	CRAE	CRVE	Fractal dimension	Tortuosity
Coronary artery calcium score	*r*= -0.085; *P* = 0.182	*r*=-0.120; *P* = 0.057	*r*=-0.055; *P* = 0.386	*r*= -0.102; *P* = 0.108	*r*= -0.108; *P* = 0.089
Average E/e’	*r* = 0.104; *P* = 0.103	*r*= -0.024; *P* = 0.702	*r*= -0.095; *P* = 0.134	*r*= -0.103; *P* = 0.106	*r* = 0.036; *P* = 0.568
LV mass/volume	*r*= -0.111; *P* = 0.078	*r* = 0.60; *P* = 0.343	*r* = 0.134; ***P*** **= 0.033**	*r*= -0.041; *P* = 0.513	*r* = 0.021; *P* = 0.739
LV global longitudinal strain	*r*= -0.088; *P* = 0.162	*r*= -0.103; *P* = 0.100	*r*=-0.42; *P* = 0.500	*r* = − 0.026; *P* = 0.678	*r* = 0.095; *P* = 0.129
Myocardial perfusion reserve	*r*= -0.040; *P* = 0.561	*r*=-0.054; *P* = 0.428	*r*=-0.021; *P* = 0.757	*r* = 0.051; *P* = 0.456	*r*= -0.094; *P* = 0.169

*Abbreviations*: AVRE = arterio-venular ratio; CRAE = central retinal artery equivalent; CRVE = central retinal venular equivalent; LV = left ventricle.

### Associations between presence of retinopathy and subclinical CVD

Logistic regression models evaluating independent associations between presence of retinopathy and pre-specified imaging criteria for subclinical CVD are presented in Table 5. Due to the low overall proportions of subjects with high LV filling pressures (average E/e’ ≥15, total *N* = 6, 2%) and reversible perfusion defects (total *N* = 21, 8%), these were not included in regression analyses. Significant independent associations between presence of retinopathy were observed with CAC ≥ 100 (OR 2.63; 95% CI 1.29–5.36; *P* = 0.008), concentric LV remodelling (OR 3.11; 95% CI 1.50–6.45; *P* = 0.002), and GLS (OR 2.32; 95% CI 1.18–4.59; *P* = 0.015). No significant associations were observed between presence of retinopathy and coronary microvascular dysfunction or presence of LGE.

**Table 5:** Associations between presence of retinopathy and subclinical CVD.

	CAC ≥ 100	LV M/V ≥ 1	LV GLS ≤ 16	MPR < 2.5	LGE present
	Odds Ratio (95% Confidence Interval)				
Age	**1.08 (1.03–1.14);** ***P*** **< 0.001**	1.06 (1.01–1.12); *P* = 0.03	0.97 (0.93–1.01); *P* = 0.20	1.03 (0.99–1.08); *P* = 0.17	1.05 (0.99–1.10); *P* = 0.11
Sex (ref. female)	**0.27 (0.14–0.50);** ***P*** **< 0.001**	0.84 (0.43–1.63); *P* = 0.61	**0.31 (0.17–0.54);** ***P*** **< 0.001**	**2.30 (1.24–4.25);** ***P*** **= 0.008**	**0.25 (0.11–0.54);** ***P*** **< 001**
Ethnicity (ref. white European)	1.00 (0.47–2.13); *P* = 0.99	0.90 (0.38–2.16); *P* = 0.81	0.75 (0.37–1.51); *P* = 0.41	1.51 (0.69–3.26); *P* = 0.30	1.04 (0.44–2.42); *P* = 0.94
Diabetes duration	1.05 (1.00-1.10); *P =* 0.06	1.02 (0.97–1.07); *P* = 0.50	1.01 (0.97–1.06); *P =* 0.62	0.99 (0.94–1.04); *P* = 0.69	1.05 (1.00-1.11); *P* = 0.07
BMI	1.05 (0.99–1.12); *P* = 0.12	1.05 (0.98–1.13); *P* = 0.14	1.00 (0.94–1.05); *P* = 0.87	0.97 (0.91–1.04); *P* = 0.40	1.05 (0.98–1.12); *P* = 0.20
Systolic blood pressure	1.00 (0.99–1.02); *P* = 0.61	0.99 (0.98–1.01); *P* = 0.54	**1.02 (1.00-1.04);** ***P*** **= 0.015**	1.01 (0.99–1.03); *P* = 0.25	1.02 (1.00-1.12); *P* = 0.08
HbA1c	1.13 (0.86–1.48); *P* = 0.39	**1.41 (1.02–1.95);** ***P*** **= 0.037**	1.11 (0.86–1.43); *P =* 0.44	1.34 (0.98–1.83); *P* = 0.06	0.74 (0.52–1.05); *P* = 0.09
Presence of retinopathy	**2.63 (1.29–5.36);** ***P =*** **0.008**	**3.11 (1.50–6.45);** ***P =*** **0.002**	**2.32 (1.18–4.59);** ***P*** **= 0.015**	0.68 (0.31–1.49); *P* = 0.34	1.26 (0.57–2.79); *P* = 0.56

*Abbreviations*: BMI = body mass index; CAC = coronary artery calcium score; GLS = global longitudinal strain; LGE = late gadolinium enhancement; LV = left ventricle; MPR = myocardial perfusion reserve; M/V = mass/volume.

### Deep learning-based prediction modelling

**Fig. 3:**
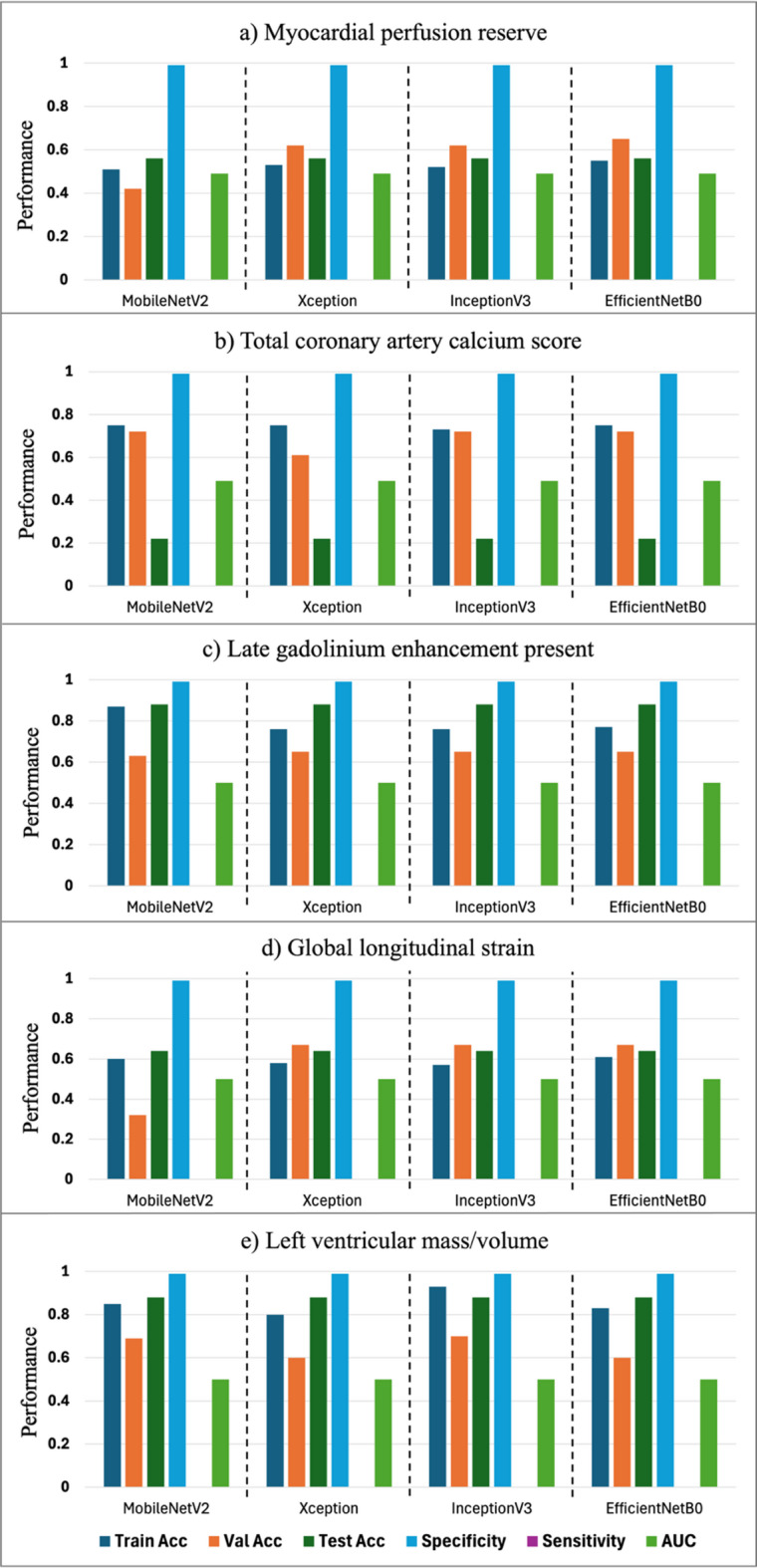
Performance of deep learning models for predicting pre-specified markers of subclinical cardiovascular disease: **(A)** Myocardial perfusion reserve (< 2.5 or ≥ 2.5), **(B)** Total coronary artery calcium score (0 or ≥ 100), and **(C)** late gadolinium enhancement (present or absent) **(D)** global longitudinal strain (< 16 or ≥ 16%), and **(E)** left ventricular mass-to-volume ratio (≤ 1 or > 1).

**Fig. 4:**
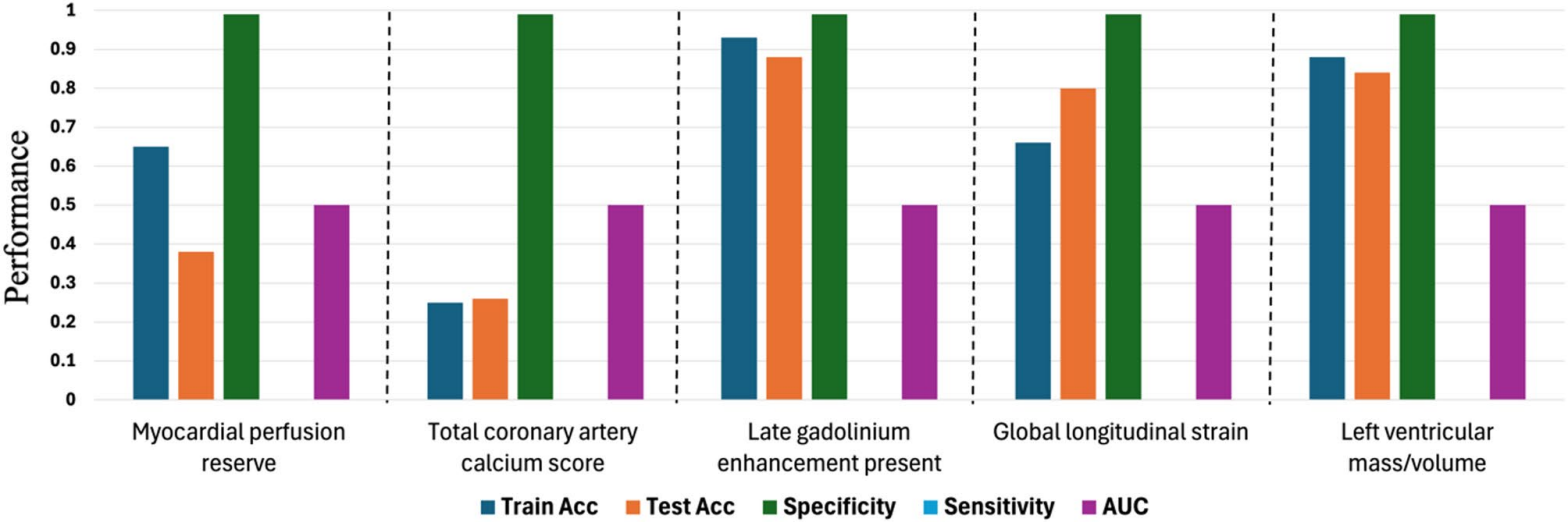
Fusion of clinical parameters with retinal photographs for detecting markers of subclinical cardiovascular disease.

Findings from the employed transfer learning models for classification of pre-specified subclinical CVD variables from digital retinal photographs are presented in Fig. 3. Despite having different architectures with respect to depth and width, similar accuracy for all models was observed. All models achieved very high specificity, reaching up to 99%. However, the sensitivity of all models was low, and resulting AUCs ~ 50%. Fusion of participant clinical characteristics with retinal images for classifying subclinical CVD did not significantly improve model performance, with AUC values remaining around 0.50 for most models (Fig. 4).

## Discussion

This study explored associations between diabetic retinopathy and retinal microvasculature geometry, with atherosclerotic CAD and markers of stage B heart failure in a cohort of people with T2D and no prior history, signs or symptoms of CVD. We found in those subjects with mild background diabetic retinopathy a strikingly greater burden of coronary atherosclerosis, more concentric LV remodelling, and diastolic and systolic dysfunction, compared to those with no diabetic retinopathy. Logistic regression analyses found strong associations between retinopathy and CAC, concentric LV remodelling, and GLS, independent of age, sex, ethnicity, diabetes duration, BMI, blood pressure and glycated haemoglobin. DL-based interrogation and quantitation of retinal microvasculature geometry did not, however, yield any correlations with relevant cardiac imaging biomarkers nor sufficient predictive performance for identifying subjects with subclinical CVD.

Several large-scale population-based studies have highlighted an elevated atherosclerotic CVD and heart failure risk associated with presence of diabetic retinopathy^5,32,33^. For example, in an analysis of 1,524 subjects with diabetes but free of prevalent CVD from the Atherosclerosis Risk in Communities cohort (age 60 ± 6 years, 47% males, 14% with retinopathy, follow-up duration 7.8 years), presence of diabetic retinopathy was associated with a twofold higher risk of incident coronary heart disease events (myocardial infarction, fatal coronary heart disease, or coronary revascularization) independent of established CAD risk factors^32^. In a separate analysis of 1,308 subjects from the same cohort, presence of retinopathy was also independently associated with a 2.5-fold increased risk of incident heart failure^5^. These studies led to suppositions that microvascular dysfunction occurs in multiple vascular beds and is a precursor to overt epicardial CAD and heart failure development in T2D^34^. However, none had comprehensively characterised the underlying cardiac alterations associated with the presence of retinopathy at baseline, as we have done in this study.

Unsurprisingly there has been growing interest in identifying direct associations between retinal vascular abnormalities and CAD. In people with T2D under evaluation for symptomatic CAD, diabetic retinopathy presence and severity was independently associated with CAD extent on invasive coronary angiography in one small study (*N* = 69)^35^, and with myocardial perfusion abnormalities on thallium-201 scintigraphy in a larger cohort (*N* = 236)^36^. More recently, and consistent with our findings in asymptomatic people with T2D, the presence of diabetic retinopathy was associated with higher CAC scores and greater prevalence of > 50% coronary stenosis on coronary computed tomography angiography^37^. However, in this cohort, most CAD cases were among subjects with moderate-severe retinopathy and no imaging markers of cardiac structure or perfusion were assessed.

To our knowledge only one study has evaluated associations between retinal vascular geometry and myocardial blood flow, albeit not exclusively in people with T2D. In a subset of 212 participants from the Multi-Ethnic Study of Atherosclerosis who underwent adenosine stress and rest perfusion CMR with myocardial blood flow quantitation and retinal photography measured vascular geometry, reductions in stress myocardial blood flow and myocardial perfusion reserve were observed with decreasing retinal arteriolar calibre, although these associations did not remain significant after adjustment for CV risk factors^38^. In our study, we found substantially higher CAC scores in those with only mild background versus no diabetic retinopathy but observed no differences in stress or resting myocardial blood flow or perfusion reserve.

Unique to our study is the incorporation of multiparametric CMR measures of cardiac structure, myocardial strain, and tissue characteristics. Coupled with transthoracic echocardiography, this enabled a comprehensive assessment for markers of stage B heart failure and their associations with retinal and kidney microvascular dysfunction as well as recommended screening tests. In our cohort, several hallmarks of stage B heart failure were evident amongst those with mild background diabetic retinopathy, including more concentric LV remodelling, elevated LV filling pressures and lower diastolic relaxation rates, and reduced GLS despite normal LV ejection fraction (with LV M/V and GLS independently associated with retinopathy). Our sensitivity analysis further demonstrated that modest variation in timing between retinal and CV assessments did not materially affect these associations, reinforcing the utility of retinal imaging as a pragmatic screening tool in routine clinical practice. These differences were observed despite groups having near identical CV risk factor profiles and no differences in serum NTproBNP or high-sensitivity troponin I concentrations. Additionally, we observed no differences in diabetic nephropathy prevalence, as serum creatinine, estimated glomerular filtration rates, and albumin-creatinine ratios were similar in participants with and without retinopathy. Our findings challenge the notion that hyperglycaemia impacts all microvascular beds equally and suggest that microvascular retinal dysfunction maybe more sensitive than microvascular kidney disease in detecting subclinical CVD. Background diabetic retinopathy may represent an early and accessible indicator of subclinical CVD in this population, offering a promising alternative to currently recommended screening approaches.

Similar associations between diabetic retinopathy and adverse cardiac structural and functional alterations (particularly increased LV mass and wall thickness, and reduced LV ejection fraction) have been reported in echocardiographic studies, although primarily in people with more severe proliferative retinopathy^39,40^. Our finding of subclinical CVD in people with T2D and only mild background diabetic retinopathy may reflect the use of advanced CMR measures with greater sensitivity than echocardiography, especially in a cohort with a high prevalence of overweight and obesity who are susceptible to poor acoustic windows with ultrasound.

Lastly, it is somewhat surprising that we found no associations between retinal vascular geometry and markers of subclinical CVD, when appraised using either a validated external retinal vessel quantification tool or the employed transfer learning models (with and without addition of clinical characteristics). We propose several explanations. First and foremost, it is possible that there is no direct link between the retinal vasculature and our pre-specified cardiac imaging parameters, precluding their prediction solely from retinal data. Related work has demonstrated high predictive accuracy in modelling age and gender from retinal images, but a marked decline in accuracy when modelling cardiac risk parameters^41^. Second, mild background retinopathy is defined by the presence of microaneurysms and/or retinal haemorrhages and may not alter the major arterial or venular morphology of the retina typically quantified by our utilised DL tools. Third, the retinal photograph images were acquired as part of routine NHS diabetic eye screening, with variations in fundus cameras, image magnification, field of view, brightness and quality that may have impacted the ability for consistent appraisal. Finally, the relatively small sample size available for our advanced computational analyses may have limited the available power for any associations to be detected. Future research should focus on improving data quality and standardisation, as well as employing advanced preprocessing techniques and integration of multimodal data, to better explore and validate the potential connections between retinal features and cardiovascular health.

### Strengths and limitations

The strengths of this study lie in its prospective design, rigorous screening and exclusion of individuals with T2D and known or symptomatic CVD, closely replicating a real-world asymptomatic screening population. Multimodality advanced cardiac imaging enabled the detection of early cardiac structural and functional alterations associated with early retinopathy. Comparable CV risk profiles in those with and without retinopathy, particularly serum NTproBNP and high-sensitivity troponin I, allowed comparisons with current screening guidelines.

Our DL models assessed prespecified imaging biomarkers of subclinical CVD rather than stage B heart failure, for which no universal diagnostic standard exists. Therefore, we could not evaluate sensitivity/specificity for stage B heart failure, which we acknowledge as a limitation. Future work should include binary outcomes to better assess diagnostic utility. Other limitations are: (1) a relatively small sample size for DL analyses, though this was mitigated using transfer learning approach to minimise the impact of our dataset size, (2) variability in retinal photograph acquisition, although these reflect real-world eye screening, (3) retinal photographs were obtained within one year of cardiovascular imaging – while same-day acquisition may have more precisely aligned with subclinical CVD status, our approach reflects routine clinical practice, maintaining practical relevance,4) the exclusion of participants with more severe grades of diabetic retinopathy, who were not available within our cohort and 5) recruitment from a single centre with a cohort predominantly of White European ancestry (76%), which may limit generalisability to more ethnically diverse populations affected by T2D and its complications. Future studies involving multi-ethnic cohorts across multiple centres are warranted to validate the applicability of these findings.

## Conclusion

In asymptomatic adults with T2D and no prevalent CVD, the presence of mild background diabetic retinopathy is associated with greater atherosclerotic burden and key markers of stage B heart failure. No direct associations between retinal microvascular geometry and subclinical CVD were observed despite using several DL algorithms. Routine diabetic eye screening may serve as a clinically relevant and accessible alternative method to currently advocated screening tools for detecting underlying CVD in T2D. These findings strengthen the existing evidence base, highlighting the need for larger, longitudinal studies to validate these results and explore the underlying mechanisms linking diabetic retinopathy and CVD. We advocate for prospective trials determining whether targeted cardiovascular screening and interventions in individuals with diabetic retinopathy can improve outcomes.

## Supplementary Information

Below is the link to the electronic supplementary material.

Supplementary Material 1

## Data Availability

The datasets used and/or analysed during the current study are available from the corresponding author on reasonable request.

## Abbreviations

AUC: area under the curve
AVRE: arterio-venular ratio
CAC: coronary artery calcium
CMR: cardiovascular magnetic resonance
CRAE: central retinal artery equivalent
CRVE: central retinal venular equivalent
CVD: cardiovascular disease
DESP: diabetic eye screening program
DL: deep-learning
GLS: global longitudinal strain
LV: left ventricle
LVM/V: left ventricular end diastolic mass-to-volume ratio
NTproBNP: N-terminal pro brain natriuretic peptide
T2D: type 2 diabetes
