# Supplementary material for: The retina as a window into detecting subclinical cardiovascular disease in type 2 diabetes
Source: Sci Rep. 2025 Jul 31;15:27968. doi: 10.1038/s41598-025-13468-4 (PMC12314106; doi:10.1038/s41598-025-13468-4)
Supplement: Supplementary file 1 — Supplementary Material 1 [file 41598_2025_13468_MOESM1_ESM.docx]

## Supplementary Tables

**Supplementary Table 1**.

| Inclusion criteria | Exclusion criteria |
| --- | --- |
| Participant is willing and able to give informed consent for participation in the study. | Angina pectoris or limiting dyspnoea (>NYHA II) |
| Male or Female, aged ≥ 18 and ≤75 years. | Major atherosclerotic disease: symptomatic CAD, history of myocardial infarction, stroke/transient ischaemic attack or symptomatic peripheral vascular disease. |
| Diagnosed with stable type 2 diabetes (determined by: i) formal diagnosis in General Practice case records, ii) a record of diagnostic oral glucose tolerance test OR glycated haemoglobin level ≥6.5%). | Atrial fibrillation or flutter. |
|  | Moderate or severe valvular heart disease. |
|  | History of heart failure or cardiomyopathy. |
|  | Type 1 diabetes mellitus (T1DM) |
|  | Low fasting C-peptide levels suggestive of adult-onset T1DM |
|  | Stage III-V renal disease (estimated glomerular filtration rate ≤30ml/min/1.73m2). |
|  | Absoloute contraindications to CMR. |

**Supplementary Table 2**. Timing of Retinal and Cardiovascular Assessments

| Interval Group | Number of Participants (N) |
| --- | --- |
| Within 0-6 months from CV assessments | 130 |
| Within 6–12 months from CV assessments | 125 |
| Median interval (days) | 51 |
| IQR (days) | -101 – 155 |

**Supplementary Table 3.** Sensitivity analysis of key cardiac imaging variables, stratified by interval between retinal photography and cardiovascular imaging (≤6 months vs. 6–12 months). Group A = ≤6 months interval; Group B = 6–12 months interval.

| Cardiac variable | ≤6M from CV assessments (N = 130) | 6–12M (N = 125) from CV assessments | P value |
| --- | --- | --- | --- |
| LV EDVi (ml/m²) | 64 ± 13 | 62 ± 13 | 0.285 |
| LV EF (%) | 67 ± 7 | 67 ± 7 | 0.814 |
| LV M/V (g/ml) | 0.91 ± 0.15 | 0.91 ± 0.14 | 0.896 |
| LV GLS (%) | 16.2 ± 2.3 | 16.3 ± 2.5 | 0.708 |
| LV circPEDSR (1/s) | 0.85 ± 0.23 | 0.86 ± 0.26 | 0.812 |
| Perfusion defect (%) | 11 (8.5%) | 10 (8.0%) | 0.890 |
| Stress MBF (mL/min/g) | 1.82 ± 0.60 | 1.83 ± 0.56 | 0.896 |
| Rest MBF (mL/min/g) | 0.65 ± 0.19 | 0.65 ± 0.18 | 0.985 |
| Total calcium score [median (IQR)] | 33 (0–206) | 38 (1–277) | 0.432 |
| CAC = 0, n (%) | 36 (28%) | 26 (21%) | 0.272 |
| CAC 1-100, n (%) | 46 (35%) | 35 (28%) | 0.206 |
| CAC 101-400, n (%) | 27 (21%) | 26 (21%) | 1.000 |
| CAC >400, n (%) | 21 (16%) | 26 (21%) | 0.452 |
